# Hypoparathyroidism in Pregnancy and Lactation: Current Approach to Diagnosis and Management

**DOI:** 10.3390/jcm10071378

**Published:** 2021-03-29

**Authors:** Dalal S. Ali, Karel Dandurand, Aliya A. Khan

**Affiliations:** Division of Endocrinology and Metabolism, McMaster University, Hamilton, ON L8S 4L8, Canada; d_alali@hotmail.com (D.S.A.); karel.dandurand@usherbrooke.ca (K.D.)

**Keywords:** hypoparathyroidism, pregnancy, calcium homeostasis, lactation, PTH/PTHrP, calcitriol, active vitamin D

## Abstract

Background: Hypoparathyroidism is an uncommon endocrine disorder. During pregnancy, multiple changes occur in the calcium-regulating hormones, which may affect the requirements of calcium and active vitamin D during pregnancy in patients with hypoparathyroidism. Close monitoring of serum calcium during pregnancy and lactation is ideal in order to optimize maternal and fetal outcomes. In this review, we describe calcium homeostasis during pregnancy in euparathyroid individuals and also review the diagnosis and management of hypoparathyroidism during pregnancy and lactation. Methods: We searched the MEDLINE, CINAHL, EMBASE, and Google scholar databases from 1 January 1990 to 31 December 2020. Case reports, case series, book chapters, and clinical guidelines were included in this review. Conclusions: During pregnancy, rises in 1,25-dihydroxyvitamin D (1,25-(OH)2-D3) and PTH-related peptide result in suppression of PTH and enhanced calcium absorption from the bowel. In individuals with hypoparathyroidism, the requirements for calcium and active vitamin D may decrease. Close monitoring of serum calcium is advised in women with hypoparathyroidism with adjustment of the doses of calcium and active vitamin D to ensure that serum calcium is maintained in the low-normal to mid-normal reference range. Hyper- and hypocalcemia should be avoided in order to reduce the maternal and fetal complications of hypoparathyroidism during pregnancy and lactation. Standard of care therapy consisting of elemental calcium, active vitamin D, and vitamin D is safe during pregnancy.

## 1. Introduction

Hypoparathyroidism (HypoPT) is a rare endocrine disorder. It is characterized by low serum calcium in the presence of a low or inappropriately normal parathyroid hormone (PTH) [1]. Phosphorus may be normal or elevated [1,2,3]. The majority of cases are secondary to neck surgery with accidental removal or injury to the parathyroid glands (75% of cases) [4,5]. The remaining 25% of cases are caused by autoimmune disorders, genetic disorders, infiltrative diseases, radiation, mineral deposition, magnesium abnormalities, and other conditions (as described in Chapter 5.1) [6,7,8]. HypoPT in pregnancy is uncommon and may pose a diagnostic challenge in early gestation due to the physiological changes in calcium and phosphorus homeostasis during pregnancy [9,10]. If not managed optimally, HypoPT may cause maternal and fetal morbidity in the form of miscarriage [11], preterm delivery, fetal skeletal demineralization [12], in utero fractures [13], fetal respiratory distress, and fetal death (as described in Chapter 6) [14]. The current literature evaluating HypoPT during pregnancy is limited to case reports and case series [9,14,15,16,17,18]. In this review, we describe calcium homeostasis during euparathyroid pregnancy and present evidence-based practical guidance on the diagnosis and management of HypoPT during pregnancy and lactation.

## 2. Materials and Methods

We carried out a literature search on the MEDLINE, CINAHL, EMBASE, and Google scholar databases from 1 January 1990 to 31 December 2020 using the following keywords: hypoparathyroidism, pregnancy, calcium homeostasis, lactation, PTH/PTHrP, active vitamin D and calcitriol. We included review articles, clinical guidelines, book chapters, case reports, and case series written in English language. Letters to the editor were excluded from the review.

## 3. Calcium and Mineral Homeostasis in Euparathyroid Pregnancy and Lactation

### 3.1. Calcium and Phosphorus

In this section, we describe the physiology of calcium regulation in pregnancy and lactation.

#### 3.1.1. Pregnancy

**Figure 1:**
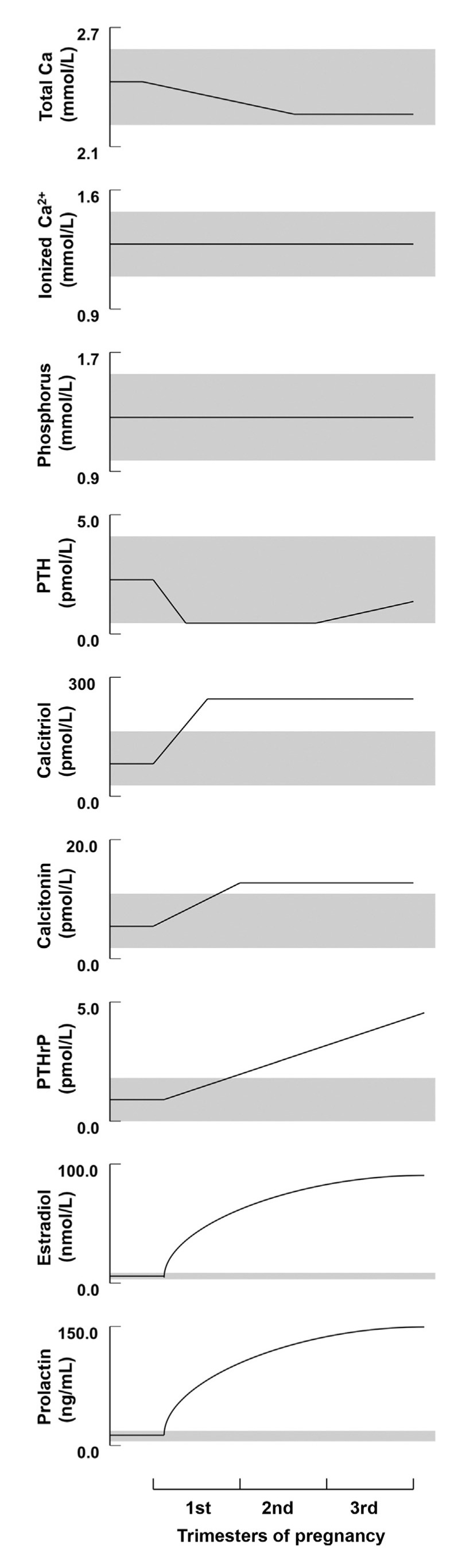
Schematic depiction of the longitudinal changes in calcium, phosphorus, and calciotropic hormone levels during human pregnancy. Shaded regions depict the approximate normal ranges (reproduced with permission form Kovacs, C.S. et al.; *Physiol. Rev.*; 2016 [20]).

Data from longitudinal and cross-sectional studies show that serum ionized calcium and calcium corrected for albumin remain unchanged during pregnancy. As albumin levels decline due to volume expansion, it is therefore essential to evaluate calcium corrected for albumin or ionized calcium for the assessment of hyper- or hypocalcemia [19,20]. Serum phosphorus and magnesium both remain unchanged during pregnancy (Figure 1) [19,20,21].

#### 3.1.2. Lactation

Serum ionized calcium or calcium corrected for albumin remain within the normal range, although may be slightly increased within the normal range, as observed in longitudinal studies [20]. Phosphorus may be increased, possibly in association with enhanced bone remodeling, favoring bone resorption with each remodeling cycle. During lactation, renal conservation of phosphorus appears to increase, despite the phosphaturic effect of PTH-related peptide (PTHrP) [22].

### 3.2. Parathyroid Hormone

#### 3.2.1. Pregnancy

Longitudinal studies from North America, Asia, and Europe have shown that PTH is suppressed into the lower end or slightly below the normal reference range in the first trimester [21,23,24,25,26]. PTH subsequently rises to the mid-normal range in the third trimester (Figure 1) [20].

#### 3.2.2. Lactation

PTHrP produced by the lactating breast suppresses endogenous PTH release; this may not happen in the presence of vitamin D inadequacy, as observed in longitudinal studies. Thus, in the presence of insufficient dietary calcium and vitamin D intake and absorption, PTH levels may not suppress [22]. Ethnic diversity may also affect PTH levels. In a prospective cohort study, African-Americans were noted to have higher levels of PTH during lactation when compared to Caucasians [27].

### 3.3. Parathyroid-Related Peptide (PTHrP)

#### 3.3.1. Pregnancy

**Figure 2:**
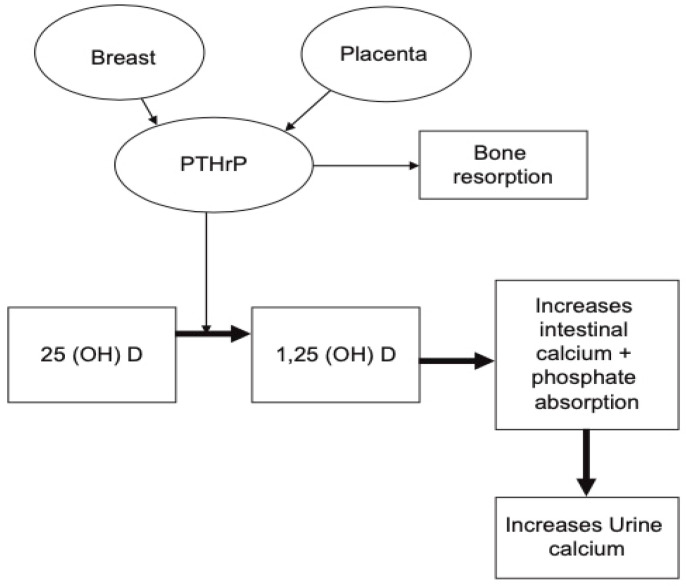
Schematic illustration demonstrating the role of PTHrP during pregnancy. It is produced by the placenta and breast tissue and can result in increase in serum calcium and phosphorus secondary to increased bone resorption and rise in 1,25-dihydroxyvitamin D. (reproduced with permission from Khan, A.A. et al.; *Eur. J. Endocrinol*.; 2019) [19].

PTHrP rises significantly and peaks in the third trimester (Figure 1). This rise has been observed in a number of longitudinal studies [21,28,29], as well as cross-sectional studies [30]. The production of PTHrP is regulated mainly by the placenta and breast (Figure 2) [19,20].

#### 3.3.2. Lactation

**Figure 3:**
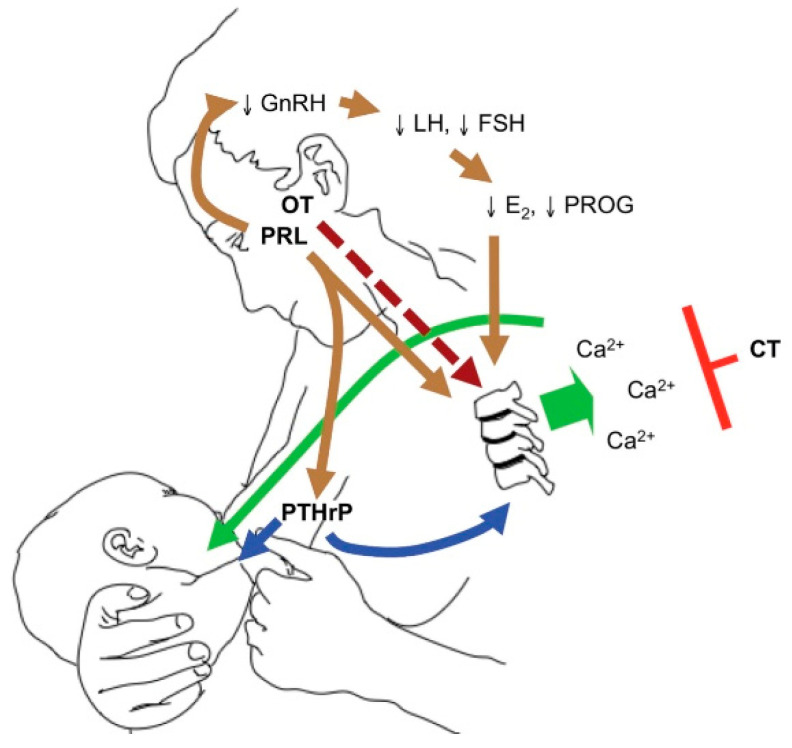
The breast–brain–bone circuit; elevated levels of PTHrP, together with low estradiol, cause bone resorption and the release of calcium and phosphorus in the circulation, which in turn reach the breast ducts and contribute to the formation of breast milk (reproduced with permission from Kovacs, C.S.; *Physiology of Calcium, Phosphorus, and Bone Metabolism During Pregnancy, Lactation, and Postweaning;* published by Elsevier: Amsterdam, The Netherlands, 2020) [22].

PTHrP produced by the lactating breast rises on suckling and reaches its maximum level at six weeks postpartum [19,31]. PTHrP levels rise with lactation, together with a fall in estradiol following delivery. These two hormonal changes influence calcium and phosphorus resorption from the skeleton to enrich the breast milk with minerals. Figure 3, by Kovacs et al., illustrates the breast–brain–bone circuit, which controls the process of lactation [22].

### 3.4. 1,25-dihydroxyvitamin D (1,25-(OH)2-D3)

#### 3.4.1. Pregnancy

The level of the 1,25-(OH)2-D3 rises by two- to three-folds in the first trimester (Figure 1) [19,32,33,34] and plays a major role in enhancing calcium absorption from the maternal gut. Evidence suggests that the maternal kidneys are the source of the 1,25-(OH)2-D3 rise during pregnancy, as well as, to a lesser extent, the placenta [19,32,35]. The expression of Cyp27b1, also known as 1-alpha hydroxylase, is increased by 35-fold in the maternal kidneys in comparison to the placenta and is possibly influenced by PTHrP, estradiol, prolactin, or placental lactogen [19,22,35,36].

#### 3.4.2. Lactation

The level of the 1,25-(OH)2-D3 falls postpartum and becomes normal during lactation. This is in association with the significant decline in estradiol levels and placental lactogen, which are believed to be the main stimulators for the 1,25-(OH)2-D3 rise during pregnancy with enhanced expression of Cyp27b1 in the maternal kidneys [22,36].

### 3.5. Calcitonin

#### 3.5.1. Pregnancy

Calcitonin is believed to rise during pregnancy [20,37,38]. Calcitonin is a polypeptide hormone that is normally synthesized by parafollicular or C cells of the thyroid gland [39]; however, in pregnancy it can be produced by the breast tissue, as well as the placenta [20]. Rises in the calcitonin level have been observed in pregnant women post-thyroidectomy, indicating that an adaptive mechanism during pregnancy is responsible for its production and release [29,40]. Calcitonin is believed to be important in protecting the maternal skeleton from increased bone resorption during pregnancy [22].

#### 3.5.2. Lactation

Calcitonin levels may rise in some women and may remain normal in others [22].

## 4. Maternal and Fetal Calcium Requirements during Euparathyroid Pregnancy and Lactation

Calcium requirements may decrease in late pregnancy, postpartum, and during lactation [41,42]. It is estimated that approximately 30 g of calcium, 20 g of phosphorus, and 0.8 g of magnesium are required by the fetus by term, with the highest rate of mineral transfer occurring in the third trimester [19,20,36,43,44]. The main sources of calcium supply to the fetus are the maternal skeleton and increased calcium absorption from the maternal gut.

The average maternal calcium deficit accrued during pregnancy and lactation is estimated to be approximately 6% of the total bone mineral content [23,45,46]. Mothers who breastfeed exclusively lose up to 10% of their bone mass [47]. Declines in bone mineral density (BMD) by 5%–10% after three to six months of lactation have been observed [20]. Data from high-resolution peripheral quantitated tomography (HR-pQCT) studies have demonstrated declines in trabecular bone thickness and cortical thickness and volume in lactating women [48,49]. Longitudinal studies involving DXA measurement in lactating women have shown recovery in BMD at 12 months postweaning [50].

## 5. Diagnosis of HypoPT in Pregnancy

Diagnosis of HypoPT is made in the presence of low calcium corrected for albumin and low or inappropriately normal PTH. During pregnancy, due to rises in PTHrP and 1,25-(OH)2-D3, the serum PTH may be even lower in an individual with HypoPT. HypoPT can be caused by a number of factors, with the commonest clinical scenario being pregnancy in a patient with postsurgical HypoPT following neck surgery; other causes are summarized in (Table 1). Careful evaluation is required to clarify the etiology as the management is affected by the underlying diagnosis, particularly in pregnancy.

### 5.1. History

A comprehensive history, including symptoms of hypocalcemia, is advised, namely, numbness or tingling in the face, hands, and feet, muscle cramping, confusion, and brain fog should be elicited. Severe symptoms of hypocalcemia may include bronchospasm, tetany, seizures, and laryngospasm, as well as congestive heart failure and arrhythmia [51]. Prior history of neck surgery and symptoms of Autoimmune Polyendocrine Syndrome type 1 (APS1), which include a history of vaginal or oral candidiasis, hypogonadism, adrenal insufficiency, type 1 diabetes, alopecia, hepatitis, and B12 deficiency, should be excluded. The presence of syndromic causes of HypoPT should be evaluated with a detailed history, including history of hearing impairment, renal impairment, or previous cardiac or oral surgeries (see Table 1 and Table 2). Other rare causes include prior exposure to ionized radiation, as well as use of certain chemotherapeutic agents such as L-asparaginase [8].

### 5.2. Physical Examination

The neck should be inspected for prior surgical incision scars. Additionally, an evaluation should be made for features of hypocalcemia and neuromuscular irritability by assessing the Chvostek’s sign by tapping the cheek 2 cm anterior to the earlobe (over the path of the facial nerve) and observing for twitching of the ipsilateral upper lip (sensitivity 25.6% and specificity 96.3%, estimated from a large cross-sectional study) [58]. Trousseau’s sign can be seen in 94% [59] of patients with hypocalcemia and is elicited by inflating the sphygmomanometer above the systolic blood pressure for a total of 3 min; carpopedal spasm indicates a positive test.

An evaluation should be made for adrenal insufficiency by checking for postural hypotension. Additionally, other clinical features of APS1 should be excluded, such as oral candidiasis, evidence of vitiligo, or hyperpigmentation. Features of syndromic causes of HypoPT should also be excluded, such as DiGeorge syndrome, Barakat syndrome, and pseudohypoparathyroidism (see Table 2) [51,53,54].

### 5.3. Laboratory Investigations

**Figure 4:**
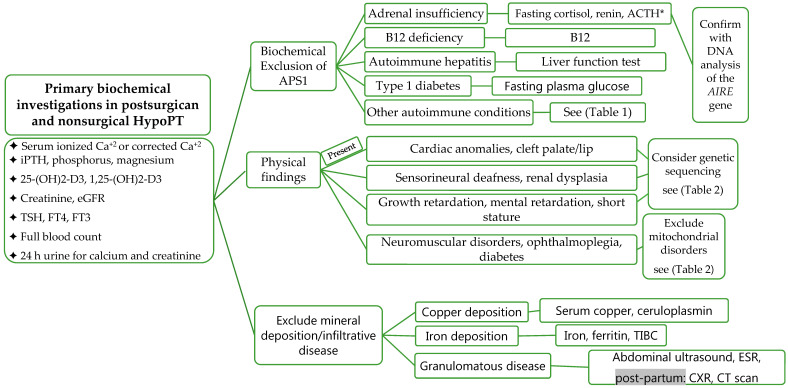
Evaluation and Investigations of HypoPT. Abbreviations: APS1, autoimmune polyendocrine syndrome; TIBC, total iron-binding capacity; Ca^+2^, calcium; eGFR, estimated glomerular filtration rate; ACTH, adrenocorticotropic hormone; CXR, chest X-ray; AIRE, autoimmune regulator of endocrine function; * please note, levels are altered during pregnancy, grey background, post-partum investigations.

Laboratory Investigations should include measurement for serum ionized calcium, calcium corrected for albumin (Corrected calcium (mmol/L) = measured total calcium + (40-serum albumin (g/L)) × 0.02. Formula (mg/dL): Corrected calcium = measured total calcium (mg/dL) + 0.8 (4.0-serum albumin (g/dL)), intact PTH (iPTH), phosphorus, magnesium, creatinine, estimated glomerular filtration rate (eGFR), 25-hydroxyvitamin D (25-(OH)2-D3), 1,25-(OH)2-D3, 24 h urine for calcium and creatinine, TSH, free T4, free T3, full blood count, copper, ceruloplasmin, iron, total iron binding capacity (TIBC), and ferritin (Figure 4). The biochemical features of APS1 should be excluded and fasting cortisol, ACTH, renin, and fasting plasma glucose should be evaluated [51,60]. During pregnancy, total and free cortisol levels, as well as cortisol binding globulins (CBG), are elevated. The hepatic CBG levels rise in response to elevated estradiol levels during pregnancy, resulting in a transient decline in free cortisol levels and a rise in ACTH [61]. ACTH is also produced from the placenta during pregnancy [61,62,63]. Maternal gonadotrophins decrease during pregnancy and become undetectable in the second trimester in response to elevated sex steroids (estradiol and progesterone) and inhibin [64].

### 5.4. Assessment of Long-Term Complications

Assessment of long-term complications should be conducted for nephrocalcinosis and nephrolithiasis by renal ultrasound. Brain imaging for basal ganglia calcification can be completed post-partum [1].

## 6. Impact of HypoPT on Mother and Fetus during Pregnancy

In HypoPT, intestinal calcium absorption is impaired due to reduced levels of 1,25-(OH)2-D3, as conversion of 25-(OH)2-D3 to 1,25-(OH)2-D3 is decreased in the absence of PTH. In pregnancy, however, 1,25-(OH)2-D3 rises, enhancing calcium absorption from the gut irrespective of PTH activity [21,65].

This adaptive mechanism may result in improvements in the serum calcium levels in HypoPT pregnant women.

It is critically important to avoid fluctuation in maternal serum calcium levels during pregnancy, as this may cause potential adverse events in the mother and fetus (Table 3). Fetal hypocalcemia can develop in the presence of maternal hypocalcemia and stimulates the fetal parathyroid glands, causing compensatory hyperparathyroidism and subsequent demineralization of the fetal skeleton [12,66,67,68] with intrauterine rib and limb fractures [12,13,14,69]. Low birth weight and intrauterine fetal death have also been reported in women with hypocalcemia during pregnancy. Maternal hypercalcemia can lead to suppression of the fetal parathyroid glands, resulting in fetal HypoPT. Neonatal hypocalcemia as a result of neonatal HypoPT may be associated with neonatal seizures and serum calcium should be checked in infants following birth [19,70]. Unexplained polyhydramnios has been reported in a case series in association with maternal hypercalcemia [71].

## 7. Clinical Management of HypoPT in Pregnancy

The impact of pregnancy on the calcium and active vitamin D required during pregnancy in women with HypoPT was recently evaluated in a case series of 12 pregnant women with HypoPT from Denmark and Canada [18]. Approximately 50% of the pregnancies included in the study required an adjustment in the dose of the active vitamin D with increases in dose required more frequently than decreases in dose. The adjustments were approximately 20% between the second and third trimesters [18]. The elemental calcium requirements were unchanged, and the serum calcium level was maintained at the lower end of the normal reference range. Other case reports, however, have reported increased elemental calcium requirements during pregnancy, especially in the first trimester [20,21,31,76]. In another case report by Sweeney et al. [41], active vitamin D requirements were reduced in the third trimester and stopped. The patient remained only on elemental calcium (1200 mg) during the third trimester.

HypoPT is a rare endocrine disorder, and recently, guidance regarding its management was published by Khan et al. [19]. Evidence has been derived from case reports and case series. The standard of care therapy for HypoPT includes calcium supplements and active vitamin D (alfacalcidiol or calcitriol or other vitamin D analogues), as well as vitamin D. It is recommended that serum ionized calcium or calcium corrected for albumin be evaluated every three to four weeks, as frequent dose adjustments may be needed according to the individual’s response [19,50]. The goal of therapy is to achieve a serum ionized calcium level or calcium corrected for albumin in the lower end of the normal reference range [19]. Only one case reported the use of rhPTH (1–34) continuous infusion via pump during pregnancy in a patient with severe hypocalcemia and recurrent hospitalizations with tetany and seizures. This patient delivered at term with no maternal or fetal complications [77]. The use of PTH in pregnancy is not approved because of lack of safety data.

## 8. Clinical Management of HypoPT during Lactation

Close monitoring of serum calcium is recommended during lactation. Postpartum hypercalcemia has been reported in lactating women with HypoPT receiving standard of care therapy (elemental calcium and active vitamin D) [78,79,80]. In one case report of a patient with HypoPT, it was possible to stop calcium and active vitamin D during lactation for 72 weeks postpartum. This patient had significantly elevated PTHrP levels from the lactating breast and was able to stop all drug therapy during the entire lactation period [78]. Postpartum women with HypoPT should be monitored, and serum calcium should be maintained in the mid- to low-normal range. Abrupt cessation of breastfeeding can be associated with hypocalcemia [17].

## 9. Conclusions

In conclusion, HypoPT requires close monitoring of serum calcium (ionized or corrected) during pregnancy and lactation, maintaining the serum calcium in the mid- to low-normal reference range in order to optimize maternal and fetal outcomes. Serum phosphorus, magnesium, and 25 (OH) D should be maintained in the normal reference range. Thiazide diuretics and PTH, both (1–34) and (1–84), should be stopped during pregnancy. Elemental calcium and active vitamin D, as well as vitamin D supplements, are safe during pregnancy. We recommend the measurement of serum ionized calcium or calcium corrected for albumin every three to four weeks during pregnancy and within one week following dose adjustment, as the half-life of calcitriol is 4–6 h and steady state is reached in five days [19]. We further recommend measuring calcium level within one-week post-partum in breastfeeding mothers as hypercalcemia may potentially result, requiring adjustment to calcium and active vitamin D supplements. 

To date, our recommendations have been based on low-quality evidence limited to case reports. The changes in calcium-regulating hormones may vary amongst individual women requiring close monitoring of serum calcium. 

Patient education regarding symptoms of hypo- and hypercalcemia should be emphasized, and patients are advised to go for immediate blood testing should symptoms occur. An individualized approach is advised in caring for pregnant women with HypoPT. A multidisciplinary team approach involves an endocrinologist, obstetrician, pediatrician, and dietitian, as well as specialized nursing staff, is essential for the achievement of optimal maternal and fetal outcomes.

## Figures and Tables

**Table 1:** Etiologies of hypoparathyroidism (HypoPT) [1,8,51,52].

Postsurgical	Autoimmune Disorders	Genetic Disorders	Radiation Exposure	Mineral Deposition	Infiltration	Magnesium Abnormality	Drugs
Most common 75% of cases post-neck surgery for thyroid cancer, laryngeal cancer, multinodular goitre, Grave’s disease, etc. Features: Presence of neck scar	♦APS1 Major features Mucocutaneous candidiasis HypoPT Adrenal insufficiency Minor features keratitis, AIH, POF, enamel hypoplasia, pneumonitis, nephritis, pancreatitis, enteropathy with chronic diarrhea or constipation, photophobia, periodic fever with rash, functional asplenia, celiac disease, type 1 diabetes, thyroiditis, retinitis, pure red cell aplasia, polyarthritis, and B12 deficiency ♦Isolated	♦DiGeorge syndrome ♦Barakat syndrome: HypoPT, deafness, renal anomaly (HDR) syndrome ♦Autosomal dominant HypoPT ♦Isolated HypoPT ♦Kenny–Caffey syndrome - Type 1 (Sanjad Sakati syndrome) - Type 2 ♦Mitochondrial disorders - Kearns Sayre syndrome - MTPD - MELAS syndrome	Exposure to ionized radiation (RAI) Features: Post-RAI therapy for thyroid cancer	♦Copper deposition (e.g., Wilson’s disease) → destruction of parathyroid gland Features: Tremors, ataxia, jaundice, psychosis, depression, and Kayser–Fleischer corneal rings ♦Iron deposition (e.g., hemochromatosis, beta-thalassemia) Features: Chronic transfusion history	Rare ♦Granulomatous disease (e.g., sarcoidosis and amyloidosis) ♦Metastasis to parathyroid gland Features: Constitutional symptoms, weight loss, and night sweats	♦Magnesium deficiency low Mg^+2^ → block cAMP in parathyroid cell, causing impaired PTH secretion Mg^+2^ activates CaSR → low Mg^+2^ → impair PTH synthesis and release ♦Hypermagnesemia high Mg^+2^ → inhibit PTH release	Chemotherapy (e.g., L asparaginase) → parathyroid necrosis Features: History of malignancy

Abbreviations: APS, autoimmune polyendocrine syndrome;; RAI, radioactive iodine; cAMP, cyclic adenosine monophosphate; MELAS, mitochondrial encephalomyopathy, lactic acidosis, and stroke-like syndrome; MTPDS, mitochondrial trifunctional protein deficiency; AIH, autoimmune hepatitis; POF, primary ovarian failure; ACTH, adrenocorticotropic hormone; TIBC, total iron binding capacity; LFT, liver function test; CaSR, calcium-sensing receptor; Mg^+2^, magnesium; →, leads to; ♦, symbol used as a bullet point.

**Table 2:** Genetic Conditions Associated with HypoPT [51,53,54,55,56,57].

Genetic Condition	Mode of Inheritance	Clinical Features	Genetic Sequencing
DiGeorge syndrome	AD	Cardiac anomalies: VSD, TOF; neurocognitive abnormalities; immune deficiency: Recurrent infections; palatal defects: Cleft palat; renal anomalies; ocular; skeletal anomalies; hearing loss	Fluorescence in situ hybridization (FISH)*, TBX1* gene sequencing
AD HypoPT ♦ADH type 1 and 2 ♦ADH type 1 with Barter’s syndrome type 5	AD	♦Type 1 and 2: Asymptomatic, mild hypocalcemia +/− hypocalciuria ♦Type1 with Barter’s: ↓Ca^+2,^ ↓Mg^+2^, ↓K^+^, hypercalciuria	♦ADH type 1—*CaSR* gene sequencing ♦ADH type 2*—GNA11* gene sequencing ♦ADH 1/Bartter’s type 5—*CaSR* gene
Barakat syndrome HypoPT, deafness, renal anomaly (HDR) syndrome	AD	Sensorineural deafness Renal dysplasia, renal failure, renal agenesis Uterine agenesis (rare)	*GATA3* gene sequencing
Isolated HypoPT	AR AD XLR	Clinical and biochemical features of HypoPT	♦AR/AD: *PTH or GCM2* gene sequencing ♦ XLR: *SOX3* gene sequencing in (males)
Kenny–Caffey syndrome ♦Type 1 (Sanjad Sakati syndrome) ♦Type 2	AR AD	♦Type 1: Short stature, dysmorphic features, growth retardation, cortical thickening of long bones, small hands and feet ♦Type2: Short stature, cortical thickening of the tubular bones, gracile bone dysplasia	♦Type 1: *TBCE* sequencing ♦Type 2: *FAM111A* sequencing
Mitochondrial disorders ♦Kearns Sayre syndrome ♦MTPD ♦MELAS syndrome	AR	♦Kearns Sayre: Ophthalmoplegia, retinal pigmentation, cardiac conduction defects, bulbar weakness. ♦MTPD: Neuropathy, retinopathy, fatty liver. ♦MELAS: Lactic acidosis, stroke-like symptoms, external ophthalmoplegia, diabetes, hearing loss, early-onset stroke symptoms	♦Kearns Sayre: *MTTL1* gene mutation ♦MTPD: *HADHA/HADHB* gene mutation ♦MELAS: *MTTL1* gene mutation (commonest)
Pseudohypoparathyroidism	AD	Short stature, brachydactyly Obesity, round facies +/– mental retardation	*GNAS* gene sequencing

Abbreviations: AD, autosomal dominant; AR, autosomal recessive; XLR, x-linked recessive; CaSR, calcium-sensing receptor; GNA11, G protein alpha subunit 11; MTTL1, mitochondrial tRNA (leucine)-1 gene; MELAS, mitochondrial encephalomyopathy, lactic acidosis, and stroke-like syndrome; MTPDS, mitochondrial trifunctional protein deficiency; Ca^+2^, calcium; K^+^, serum potassium; VSD, ventricular septal defect; TOF, tetralogy of Fallot, Mg^+2,^ magnesium; ↓, low; +/−, with or without; ♦, symbol used as a bullet point.

**Table 3:** Reported maternal and fetal adverse events in HypoPT [8,11,13,17,18,36,70,71,72,73,74,75].

Maternal Serum Calcium	Fetus	Mother
High Maternal Ca^+2^	Hypoparathyroidism, polyhydramnios, and neonatal seizures	Hypercalciuria and kidney stones
Low Maternal Ca^+2^	Hyperparathyroidism, increased bone resorption, intrauterine fragility fractures, subperiosteal bone resorption, osteitis fibrosa cystica, and respiratory distress	Miscarriage, preterm labor, seizure, and arrhythmia

## Data Availability

Not applicable. No new data were created or analyzed in this study.
